# Results from an exploratory study to test the performance of EQ-5D-3L valuation subsets based on orthogonal designs, and an investigation into some modeling and transformation alternatives for the utility function

**DOI:** 10.1186/s13561-014-0029-z

**Published:** 2014-11-08

**Authors:** Henry Bailey, Paul Kind, Althea La Foucade

**Affiliations:** Arthur Lok Jack Graduate School of Business, The University of the West Indies, St. Augustine Campus, Max Richards Drive, Uriah Butler Highway, Champs Fleurs, Trinidad and Tobago; HEU, Centre for Health Economics, The University of the West Indies, St. Augustine Campus. 25A Warner Street, St. Augustine, Trinidad and Tobago; Academic Unit of Health Economics, Institute of Health Sciences, University of Leeds, Leeds, LS2 9LJ United Kingdom

**Keywords:** Valuation subset, Orthogonal array, EQ-5D, Visual analogue scale

## Abstract

**Background:**

EQ-5D-3L valuation studies continue to employ the MVH protocol or variants of MVH. One issue that has received attention is the selection of the states for direct valuation by respondents. Changes in the valuation subset have been found to change the coefficients of the utility function. The purpose of this study was to test the performance of valuation subsets based on orthogonal experiment designs. The design of the study also allowed a comparison of models based on raw or untransformed VAS values with values transformed at the level of the respondent and at the aggregate level.

**Methods:**

Two different valuation subsets were developed based on orthogonal arrays. A VAS elicitation was undertaken with two groups of similar respondents and the resulting utility functions based on the valuations of the two different valuation subsets were compared using mean absolute errors between model and observed values, and by correlation with values in and out of sample. The impact of using untransformed versus VAS values transformed at the level of the individual and at aggregate level and the inclusion of a constant term in the utility functions were also investigated.

**Results:**

The utility functions obtained from the two valuation subsets were very similar. The models that included a constant and based on raw VAS values from the two valuation studies returned rank correlation coefficients of 0.994 and 0.995 when compared with respective observed values. MAEs of model values with observed values were 2.4% or lower for all models that included a constant term. Several models were developed and evaluated for the combined data (from both valuation subsets). The model that included the N3 term performed best.

**Conclusions:**

The finding that two very different valuation subsets can produce strikingly similar utility functions suggests that orthogonal designs should be given some attention in further studies. The impact of rescaling VAS values at the level of the individual versus at aggregate level had minimal impact on the performance of the models when compared to models based on the raw VAS values.

## Background

Health states defined by EQ-5D and other health status classification systems such as HUI [1] and SF-6D [2] are typically represented by a summary index score computed once the value of different dimensions and levels within dimensions have been established. Studies that generate such value sets for these instruments often adopt a similar approach in order to overcome the respondent burden involved in assessing large numbers of health states. In the case of EQ-5D-3L a total of 243 health states are defined by the descriptive classification, there being 5 dimensions (mobility, self care, usual activities, pain/discomfort and anxiety/depression) for each of which there are 3 possible responses levels. The response level for each dimension is used to create a numeric code that acts as a nominal descriptor for each state. The logically best health state is coded as 11111 (no problem on any of the 5 dimensions); the logically worst health state is coded as 33333 (an extreme problem on all 5 dimensions). It is usually the case that a smaller number of selected health states are presented for direct evaluation in any valuation study. These directly observed values are then used to construct a statistical model from which to estimate the value decrements associated with each dimension/level. These derived values are then applied to compute index scores for the full set of health states defined by the classification system.

Valuation studies have taken several approaches when selecting a subset of EQ-5D states for direct assessment. The first large EQ-5D valuation study was the Measurement and Valuation of Health (MVH) study in the United Kingdom carried out in 1993 [3].

The MVH protocol used a subset of 43/243 EQ-5D states plus unconscious and immediate death (a total of 45 states). Valuation of all states in this subset was considered to be too much by way of respondent burden and a block design was used so that each respondent evaluated a total of 15 states. The reduction of respondent burden in this way necessitated an increase in the size of the study sample. Subsequent interest in identifying efficient subsets for EQ-5D valuation studies has yielded a number of alternative designs.

The valuation subset used in the MVH study comprised 43 states that were selected to cover a wide range of severity, to maintain consistency with an earlier study that had been conducted in Finland, and to include only states that would be considered by the researchers to be ‘plausible’ to the average respondent [4]. As an example of the ‘plausibility’ criterion, any states that combined level 3 on Mobility (confined to bed) with level 1 on ability to perform Usual Activities or Self Care (that is all states comprising 3X1XX or 31XXX) were excluded.

One study [5] that specifically set about to investigate the performance of valuation sets for EQ-5D evaluated several subsets of states used in the MVH study by testing the performance of models in terms of correlation between observed and predicted values and Mean Absolute Error (MAE). This study used a backward sequential elimination algorithm to remove the state at each step with the smallest effect on the regression models. A final subset of 17 states referred to as the Macran and Kind States performed best on the correlation criteria. These analyses were conducted on the MVH data.

In another study that was based on MVH data, Lamers et al. [6] used simulated sampling strategies to model the performance of various subsets of the MVH valuation set. The resulting models were compared in terms of correlation and MAE with observed values. This approach was not able to identify a valuation subset that outperformed the Macran and Kind Set.

Zarate and Kind [7] attempted to identify smaller valuation subsets in other countries that had conducted EQ-5D-3L valuation studies using the MVH valuation set. This approach was taken for data from the USA, Chile and the UK. In all three cases, the minimum number of states in the valuation subset that could be kept while avoiding a ‘large’ increase in MAE versus observed values was 17. Removal of further states from the valuation set resulted in MAE of the model values versus observed values moving from 0.05 to over 0.1 (on a 0–1 scale) in all three cases. The problem was that the identity of the 17 states differed between the three countries in the study. This suggests that a single small (eg 17 state) valuation subset that can be applied to all countries may not exist however a common set of 31 states was found which may perform reasonably well when applied to the data for the three countries in the study. These studies suggest that the states comprising the valuation subset affect the model that is obtained.

Other EQ-5D valuation studies have used the Macran and Kind subset combined with 8 other states drawn randomly from the rest of the MVH set [8,9].

A large valuation subset comprising 101 states was used in South Korea [10]. Using large valuation subsets may improve precision since this leaves fewer states that must be valued based on modeling. However, using large valuation subsets also increases the number of respondents required in valuation studies, since blocking methods must be used to break the subset into smaller components for valuation by individual respondents.

Bagust [11] attempted to build a valuation subset using a set of four explicit quantifiable criteria:

1. Plausibility: by examining large empirical data sets to find states that are observed in the population for which the value set is being developed.
2. Relevance: the states selected for direct valuation should be those most frequently reported by the population for which the value set is being developed.
3. Coverage over severity range: This is related to the ‘code score’ of an EQ-5D state which is obtained by adding the value of the level of each dimension in the state. Thus state 11111 has a code score of 5 × 1 = 5, and state 12223 would have a code score of 1 + (3 × 2) + 3 = 10. The state that lies furthest from 11111 is 33333 which has a code score of 5 × 3 = 15. This measure gives a general indication of severity, so a valuation set based on this approach would include states covering all possible levels of code score from 5 to 15.
4. Simple severity increments: valuation subsets should comprise states that represent single ‘adjacent steps’ (i.e. states having a difference in code score of 1) in progressing from 11111 to 33333. It is argued that this would allow direct measurement of the lowest level of differentiation that can be obtained from the EQ-5D-3L system.

This approach produces a set of 55 states in 5 blocks, so that each respondent values a sub-set of 11 states. The study developed a valuation subset based on MVH data, but there is no application or empirical data regarding the performance of this valuation set. The approach would require the self reported EQ-5D states for thousands of citizens of the country for which a value set is being developed in order to identify the states that meet criteria 1 and 2. Most developed countries now have EQ-5D Value Sets [12]. Moving forward, it is expected that ‘new’ countries for which EQ-5D-3L value sets are to be developed will comprise middle income or developing countries for which self reported health for such large numbers of citizens will not be available. This approach also requires 5 respondents per replicate.

The purpose of our study was to test the performance of valuation subsets based on orthogonal experiment designs. An orthogonal design is one in which the columns of the independent variables are orthogonal to each other. For the design of an EQ-5D valuation subset, this would mean that in each replicate, each level of every dimension would appear an equal number of times. Historically, orthogonal experiment designs have been used extensively in many fields [13]. For this study, a Visual Analogue Scale (VAS) was used to capture the observed values.

The design of this study also allowed the opportunity to test two further issues involved in modeling EQ-5D valuation data. These are 1- the question of whether to transform the VAS values (on to a 0 to 1 scale) at the level of the individual, or at the aggregate level, and 2- the effect of the inclusion of a constant term and additional dummy variables in the regression model.

In order to derive weights that would enable EQ-5D states to be used as quality-adjustment scalars in QALY calculations, the data must be rescaled using a monotonic transformation to map the Visual Analogue Scale (VAS) values of states on a 0 to 1 scale, corresponding to the states dead and full health respectively. One approach is to carry this transformation out at the level of the individual respondent using an adjustment:

$$ \mathrm{V}\mathrm{A}{\mathrm{S}}_{\mathrm{Rescaled}}=\left[\mathrm{V}\mathrm{A}{\mathrm{S}}_{\mathrm{Raw}}\hbox{--} \mathrm{D}\mathrm{e}\mathrm{a}{\mathrm{d}}_{\mathrm{Raw}}\right]\div \left[1111{1}_{\mathrm{Raw}}\hbox{--} \mathrm{D}\mathrm{e}\mathrm{a}{\mathrm{d}}_{\mathrm{Raw}}\right] $$

The same form of transformation could be applied to aggregated observed data taking the mean value, for example, as the measure of central tendency. The advantage of this approach is that it effectively dampens the effect of variability within an individual respondent’s data thereby introducing a degree of smoothing and potentially giving rise to a simplified estimation model. On the other hand, this approach could be criticized for losing some of the individual data. To compare these two approaches, regression analyses were run on VAS values transformed at the level of the individual and at aggregate level. The regression analyses were also run on the raw or untransformed VAS values to allow for comparison with the models based on transformed VAS values.

A further element of consideration in developing estimation models was the use of a constant term. Many EQ-5D valuation studies include a constant term which is interpreted behaviorally as representing the value decrement accounted for by any departure from full health [14]. However, the impact of including such a constant term has not been the subject of any systematic investigation. Its inclusion seems to be a consequence of adopting previous custom and practice rather than being a deliberate choice. The use of a constant term may mask imperfections in the specification of the model and/or the volume of information under investigation and its use may simply be to act as a proxy for unobserved variance not otherwise specified. Alternate models were developed in this study in which the regression lines were forced through the origin. These models were considered and evaluated as counterfactuals to the models in which the constant term was permitted.

## Methods

**Figure 1:**
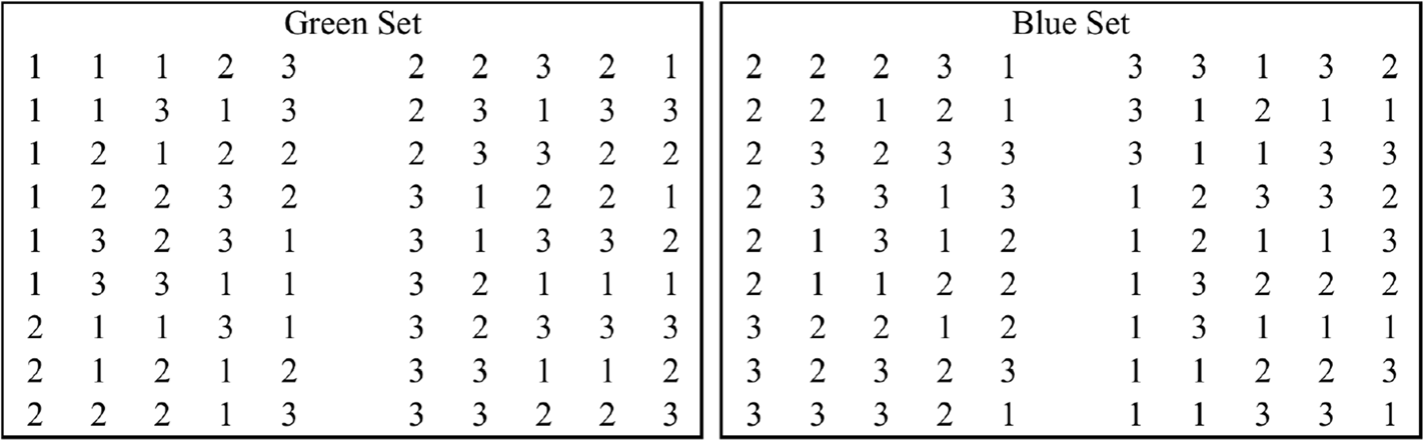
**Valuation subsets.**

Two valuation subsets were designed, the first of which (labeled ‘Green Set’) was created using a pre-existing orthogonal array [15] and is shown in Figure 1. The second (labeled ‘Blue Set’) was derived from the first by adding generator 11111 to each member of the first orthogonal array using modulo 3 arithmetic. EQ-5D states 11111, 33333 and ‘Dead’ were added to each set as anchors, along with two further ‘hold-out’ states which were not included in the regression analysis but were used to evaluate models produced by the analysis. The total number of states valued by each responded was 23, being the 18 states based on one of the orthogonal arrays plus these 5 additional states (11111, 33333, ‘Dead’ and the two holdout states).

A sample of 230 university students took part in a valuation exercise conducted at the St. Augustine campus of the University of the West Indies. All of the elicitations were conducted in a 1:1 office setting and respondents received TT$50.00 (equivalent to US$8) at the start of the interview. Respondents were randomly assigned to the Green and Blue valuation sets.

**Figure 2:**
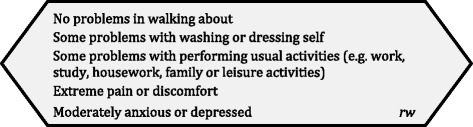
**Example of an EQ-5D-3L Ranking/VAS card.**

Respondents were first asked to complete the standard EQ-5D self-classifier that records their own assessment of their health status and was designed as a ‘warm up’ task to build familiarity with the descriptive system in original EQ-5D valuation studies. Upon completion respondents were handed a set of cards containing descriptions of EQ-5D health states. The cards were 4 cm × 12 cm in size with protruding rhomboid edges that made them hexagonal as is shown in Figure 2. Each respondent was randomly assigned to one of two ‘Green’ and ‘Blue’ sets.

Each card had a two letter code printed in the bottom right corner so that the interviewer could record the rank data and VAS valuations. It was explained to the respondents that these codes were generated randomly and had no significance.

The cards were ranked from best to worst along the edge of a desk with respondents first being handed two randomly selected cards and instructed to place one card on the desk and decide whether the second was better or worse than the first, placing it above or below accordingly. A third card was then introduced and the respondent was asked to decide whether this should go above, below or in between the other two. This process was repeated until all 23 cards for that respondent were ranked. Tied ranks were permitted.

Once this ranking task was complete the interviewer noted the order of health states and then placed a 1-metre version of the VAS alongside the ranked cards. Respondents adjusted the location of each card so that the rhomboid edge pointed to the VAS rating corresponding to their assessment of the value of each state on the 0 – 100 scale. This allowed the respondent to see all of the cards on the VAS at the same time and to adjust their positions and values. Respondents were reminded that ties were permitted and that they had the freedom to change the order of states if they so chose. Interviewers had been instructed that if a respondent raised the issue of an implausible state, they were to respond with a statement explaining that some people do find that some of the states are difficult to imagine and to encourage respondents to carry out the valuation (or ranking task) for the state to the best of their ability. Once the VAS task was finished the interviewer recorded the rating scores for all health states.

**Figure 3:**
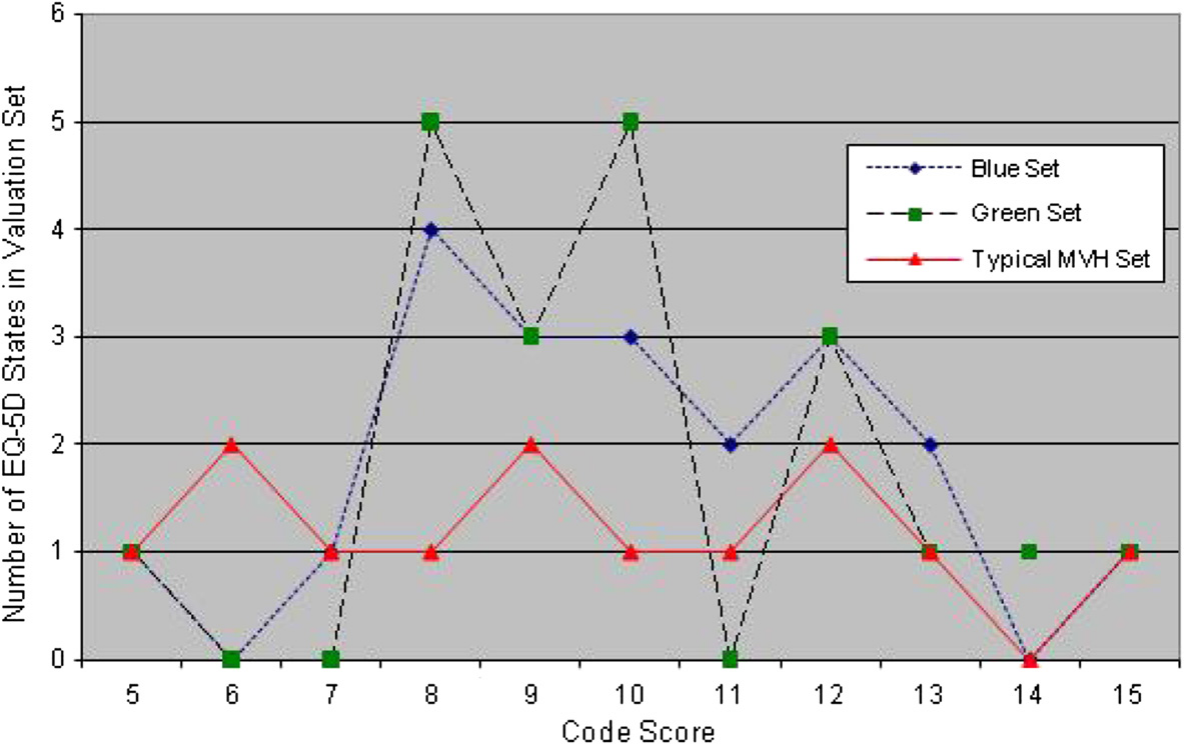
**A Comparison of the orthogonal valuation sets with a typical MVH set by code scores.**

The use of orthogonal subsets can present special challenges to the respondent when compared to subsets designed in other ways, such as those used in the MVH study. The MVH study sought to avoid implausible states, however these are an inevitable feature when using an orthogonal array that brings every level of every dimension together with every level of every other dimension. Here implausible states cannot be avoided—for example states combining ‘confined to bed’ with ‘no problems’ on the ‘Self Care’ and ‘Usual Activities’ dimensions were observed in both the Green and Blue valuation subsets. Further, the MVH valuation subsets are balanced with 2 or 3 states in progressively more “severe” categories (mild, moderate and severe states). The orthogonal sets are more concentrated with more than half of the states in the moderate code score range of 8–11, so that preferences among the states are less obvious to respondents using the orthogonal sets (Figure 3). Generally, respondents would be expected to have greater difficulty in assigning ranks and values among sets of states that are more similar in severity/code score than among sets in which states that are further apart in terms of severity [16].

### Analysis

Although panel regression methods would generally be appropriate, the analysis of pooled data from an orthogonal experiment design using ordinary least squares (OLS) regression produces identical coefficients to fixed and random effects models [17]. Given that the valuation sets used in this study were based on orthogonal arrays, the models were produced using OLS. All regression analyses were carried out using Stata Statistical Software 12.0.

In the absence of having access to a ‘true’ underlying utility function, the models obtained in EQ-5D valuation studies are usually evaluated on such criteria as internal validity, Mean Absolute Error (MAE) versus observed values, R-Squared etc. In addition to these criteria, this study design allowed for a comparison between the utility functions based on valuations by two groups of similar respondents, using two completely different valuation subsets (with no states in common) that were both developed from orthogonal arrays.

Statistical analysis was conducted in two stages. In the first stage, 6 different model specifications were used to analyse the two respondent data sets (Blue and Green) as follows:

- Model 1: with the dependent variable as 100 – raw VAS value with no constant term in the model.
- Model 2: with the dependent variable as the raw VAS value with a constant term in the model.
- Model 3: with the dependent variable as 1 –VAS value rescaled at the level of the individual respondent with no constant term in the model.
- Model 4: with the dependent variable as the VAS value rescaled at the level of the individual respondent with a constant term in the model.
- Model 5: with the dependent variable as 1 –VAS value rescaled at aggregate level using mean values with no constant term in the model.
- Model 6: with the dependent variable as the VAS value rescaled at aggregate level using mean values with a constant term in the model.

Testing models 3 through 6 allowed a comparison of the performance of models with and without the constant term, as well as with VAS values transformed at the level of the respondent and at aggregate level. Including models 1 and 2 allowed a comparison of the models with equivalent analyses based on untransformed VAS values. Each model was evaluated using the following criteria - adjusted R^2^, within-sample correlation, correlation with out-of-sample values, MAE of estimated and observed values, and the percentage of model versus observed residuals that were above 5% (i.e. residuals above 5 VAS points for the Raw VAS models and residuals above 0.05 for the Rescaled models).

In the second stage of the analysis, the best performing model using rescaled data based on these criteria was used to develop a model for the pooled data (combining the Blue and Green observed values). The performance of this model was compared with variants of this baseline model that included dummy variables which had previously been specified in the other valuation studies. These dummy variables indicated the presence of any 1’s, 2’s and 3’s in a state (N1, N2 and N3 respectively). In addition to these, regressions were also run with dummy variables giving the numbers of 1’s, 2’s and 3’s in a state (C1, C2 and C3 respectively) and the squares of these counts (C1Sq, C2Sq, C3Sq). These regressions were run to test whether these additional variables would improve performance over the baseline model.

These models were compared based on adjusted R^2^, correlation of model values with observed values, and MAE of model values and values observed for the sample and hold out states.

## Results

230 individuals participated in this study. The median age was 20 and 40% were male. The rates of self-reported problems as shown in Table 1 vary across the EQ-5D dimensions with only 1 respondent recording any problem with self-care compared to 90 respondents (=39%) who reported a problem with anxiety/depression. The self-reported health status as indicated by the mean of their VAS ratings was 76.1 (s.d. =13.5).

**Table 1:** **Problem rates reported by the respondents** ^**a**^

	**% of Respondents**	
	**Level 2**	**Level 3**
Mobility	2.6%	0.4%
Self-care	0.4%	0.0%
Usual activities	12.2%	0.0%
Pain/Discomfort	36.5%	0.9%
Anxiety/Depression	33.5%	5.7%

Valuation data for the two sets of 23 states were obtained from 112 (Blue) and 117 (Green) respondents respectively. VAS values were obtained for 38 EQ-5D states plus dead. The mean observed values and corresponding transformed scale values are given in Table 2.

**Table 2:** **Mean observed and rescaled VAS values** ^**b**^

**State**	**Set**	**Observations**	**VAS untramsformed**		**VAS rescaled individual**		**VAS rescaled aggregate**	
			**Mean**	**St. dev**	**Mean**	**St. dev**	**Mean**	**St. dev**
11111	Both	229	97.5	6.3	1.0000	0.0000	0.9828	0.0474
11123	Green	112	73.1	21.3	0.7404	0.2584	0.7461	0.2221
12122	Green	112	68.0	20.7	0.6828	0.2359	0.6928	0.2157
11313	Green	112	66.6	19.1	0.6794	0.2301	0.6781	0.1990
13111	Blue	117	66.5	20.3	0.6716	0.2086	0.6774	0.2115
21212	Green	112	64.3	19.3	0.6506	0.2314	0.6540	0.2010
21122	Blue	117	63.8	16.5	0.6494	0.1707	0.6486	0.1715
11223	Blue	117	61.4	22.5	0.6255	0.2317	0.6242	0.2343
12113	Blue	117	58.9	21.7	0.6012	0.2236	0.5981	0.2264
22121	Blue	117	58.7	18.3	0.5979	0.1858	0.5954	0.1901
13311	Green	112	57.5	23.3	0.5799	0.2390	0.5834	0.2421
11331	Blue	117	57.1	19.0	0.5784	0.1975	0.5786	0.1979
21131	Green	112	56.4	19.8	0.5678	0.2181	0.5716	0.2058
21312	Blue	117	56.0	18.2	0.5651	0.1877	0.5679	0.1891
12232	Green	112	52.8	22.1	0.5284	0.2434	0.5340	0.2302
13222	Blue	117	51.1	20.1	0.5179	0.2072	0.5163	0.2089
22213	Green	112	49.3	17.4	0.4947	0.2090	0.4983	0.1820
22321	Green	112	48.9	17.8	0.4898	0.2029	0.4939	0.1853
13231	Green	112	48.1	23.6	0.4771	0.2446	0.4857	0.2457
12332	Blue	117	46.6	19.3	0.4707	0.2017	0.4696	0.2008
22231	Blue	117	43.9	18.1	0.4431	0.1859	0.4415	0.1888
23322	Green	112	40.0	19.0	0.3940	0.2152	0.4010	0.1978
31211	Blue	117	37.1	22.1	0.3717	0.2335	0.3709	0.2305
23313	Blue	117	36.8	18.9	0.3686	0.2077	0.3678	0.1970
32111	Green	112	33.1	20.5	0.3214	0.2406	0.3297	0.2134
23133	Green	112	31.0	18.8	0.2969	0.2198	0.3069	0.1960
32212	Blue	117	29.8	19.0	0.2960	0.2022	0.2953	0.1974
31221	Green	112	29.7	18.8	0.2864	0.2291	0.2939	0.1956
23233	Blue	117	28.1	17.7	0.2801	0.1841	0.2774	0.1846
33112	Green	112	23.9	16.8	0.2254	0.2141	0.2337	0.1747
31133	Blue	117	22.6	16.5	0.2193	0.1779	0.2196	0.1718
33132	Blue	117	19.4	14.9	0.1855	0.1671	0.1796	0.1363
33321	Blue	117	19.0	13.3	0.1831	0.1507	0.1824	0.1383
31332	Green	112	18.7	13.1	0.1730	0.1746	0.4415	0.1888
32323	Blue	117	18.0	12.6	0.1728	0.1397	0.1718	0.1316
33223	Green	112	16.3	13.3	0.1457	0.1801	0.1548	0.1387
32333	Green	112	12.0	11.7	0.0986	0.1684	0.1091	0.1221
33333	Both	229	7.0	9.1	0.0463	0.1136	0.0508	0.0888
Dead	Both	229	1.5	6.1	0.0000	0.0000	0.0107	0.0503

Results of the first stage are presented in Table 3. The models based on VAS values transformed at the aggregate level and excluding the constant term had higher adjusted R^2^ values than the other models. However on the criteria that deal with predictive ability (MAE and correlation measures) the models that included the constant term outperformed those that exclude it for both the green and the blue set. Comparing the models based on the VAS values transformed at the two levels that include the constant term, Model 6 has a higher adjusted R^2^, but Model 4 has better MAE and correlation measures. These differences are very small.

**Table 3:** **Results of the first stage analysis for the blue and green sets**

**GREEN SET**	**Model 1**	**Model 2**	**Model 3**	**Model 4**	**Model 5**	**Model 6**	
			**VAS rescaled**				
	**VAS raw**		**Individual level**		**Aggregate level**		**Model 4 versus Model 6**
	**No constant**	**With constant**	**No constant**	**With constant**	**No constant**	**With constant**	
Adjusted R-Sq	0.5660	0.5988	0.5281	0.5489	0.9	0.6	−0.0499
% MAE: Green Model w/ Green Observed	5.50%	2.40%	5.08%	2.38%	5.01%	2.35%	0.03%
% Residuals >5% (i.e. 5.0 for Raw, and 0.05 for Rescaled)	55%	5%	50%	10%	50%	5%	5%
% MAE: Green Model w/ Blue Observed	6.50%	3.90%	6.30%	4.08%	6.13%	4.59%	−0.51%
% MAE: Green Model w/ Green Holdouts	5.15%	4.65%	5.28%	4.84%	6.03%	5.61%	−0.77%
Correlation within sample (Green Model w/ Green Observed)	0.9778	0.9945	0.9804	0.9943	0.98	0.9940	0
Correlation out of sample (Green model w/ Blue Observed)	0.9725	0.9871	0.9731	0.9854	0.97	0.97	0.01
	**Model 1**	**Model 2**	**Model 3**	**Model 4**	**Model 5**	**Model 6**	
**BLUE SET**			**VAS rescaled**				
	**VAS raw**		**Individual level**		**Aggregate level**		**Model 4 versus Model 6**
	**No Constant**	**With Constant**	**No Constant**	**With Constant**	**No Constant**	**With Constant**	
Adjusted R-Sq	0.5280	0.57	0.54	0.57	0.9	0.57	0
% MAE: Blue Model w/ Blue Observed	5.40%	2.25%	5.00%	2.25%	4.90%	2.21%	0.04%
% Residuals >5% (i.e. 5.0 for Raw, and 0.05 for Rescaled)	40%	10%	40%	10%	40%	10%	0%
% MAE: Blue Model w/ Green Observed	6.37%	3.85%	5.99%	3.81%	6.25%	3.85%	−0.04%
% MAE: Blue Model w/ Blue Holdouts	4.53%	2.46%	4.22%	2.63%	5.95%	2.62%	−0.01%
Correlation within sample (Blue Model w/ Blue Observed)	0.9725	0.9944	0.9745	0.9936	0.9750	0.99	0
Correlation out of sample (Blue model w/ Green Observed)	0.9594	0.9818	0.9631	0.9821	0.9630	0.9820	0

**Figure 4:**
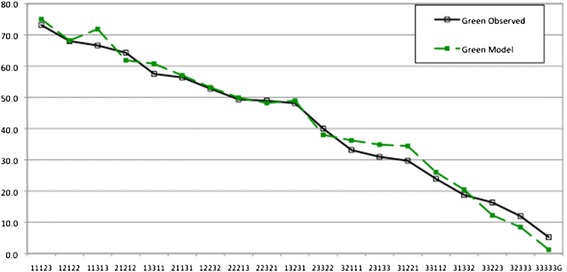
**VAS values from the green model (raw VAS with constant term) compared with observed VAS values from the green set.**

**Figure 5:**
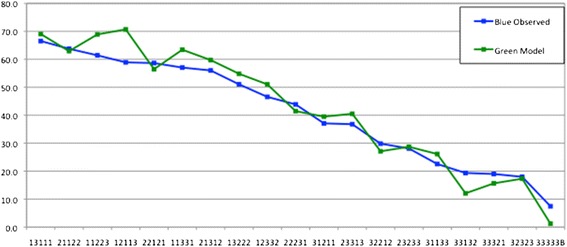
**VAS values from the green model (raw VAS with constant term) compared with observed VAS values from the blue set.**

Figures 4 and 5 show the performance of the model based VAS values from the Green data set (Model 2) compared to the observed values from the Green and Blue sets.

**Figure 6:**
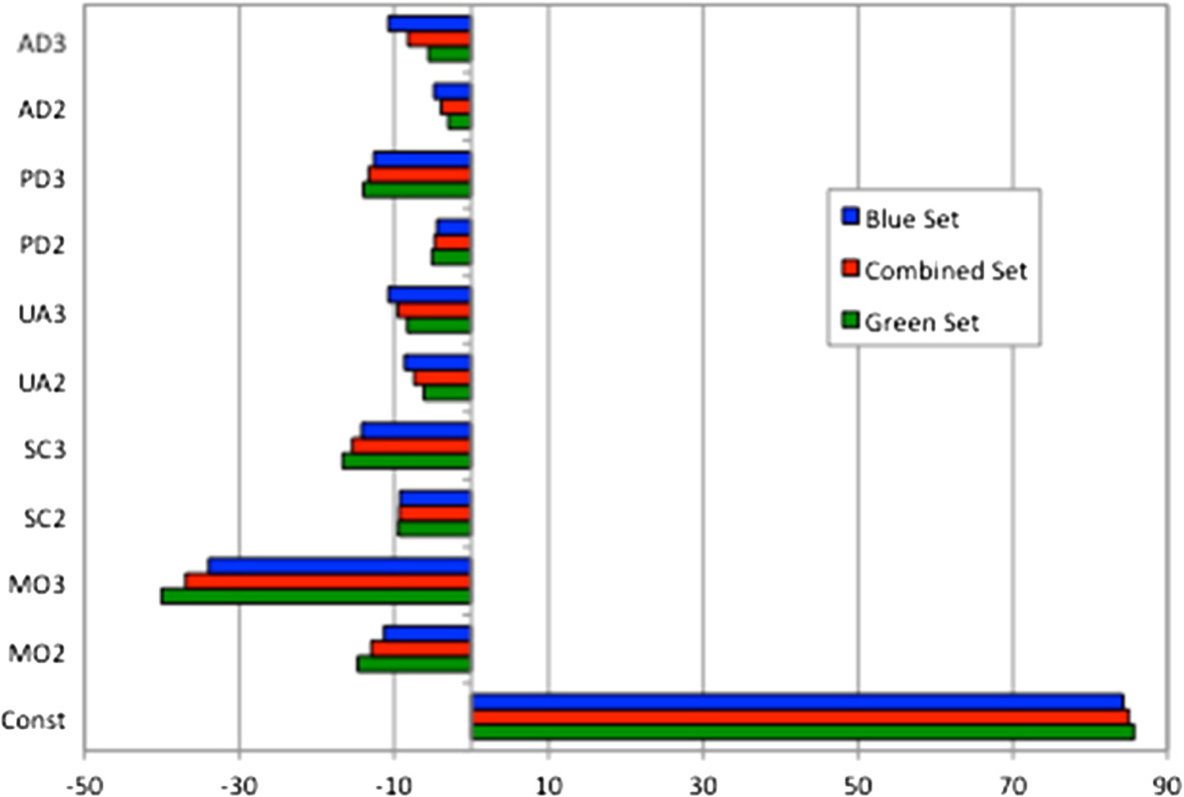
**Comparison of the coefficients of model 2 from the green, blue and combined data sets.**

The models based on the green and blue data sets comprise very similar coefficients. This similarity is displayed in Figure 6 where the coefficients for the models based on raw VAS values from the two data sets (Model 2) are compared. This pattern of very similar coefficients also follows for models based on data transformed at both levels.

The effect of including additional dummy variables was investigated using Model 4 for the combined data set (green plus blue). Table 4 shows the value decrements for each dimension/level using a baseline regression model that utilises VAS values transformed at the level of the individual and including a constant term. Only models that were internally valid are displayed (the C1, C2, C3, C1Sq and C2Sq models all included counterintuitive coefficients with level 2 having a higher absolute decrement than level 3 for one or more dimensions). The N3 model outperformed the other 4 models in Table 4 on all five criteria that were used for this comparison (R^2^ as well as the predictive measures). The magnitude of these improvements in performance is however small.

**Table 4:** **Comparison of the 5 models based on the combined set**

	**Baseline model**	**N1**	**N2**	**N3**	**C3Sq**
**Constant**	0.8685	0.9830	0.8865	0.9279	0.8921
**MO2**	−0.1350	−0.1437	−0.1084	−0.1442	−0.1395
**MO3**	−0.3874	−0.4061	−0.3725	−0.3749	−0.4325
**SC2**	−0.0975	−0.1061	−0.0766	−0.0876	−0.0911
**SC3**	−0.1614	−0.1802	−0.1581	−0.1394	−0.2023
**UA2**	−0.0764	−0.0850	−0.0613	−0.0475	−0.0674
**UA3**	−0.0988	−0.1176	−0.1070	−0.0673	−0.1322
**PD2**	−0.0495	−0.0650	−0.0289	−0.0587	−0.0523
**PD3**	−0.1390	−0.1510	−0.1362	−0.1265	−0.1807
**AD2**	−0.0410	−0.0426	−0.0202	−0.0494	−0.0420
**AD3**	−0.0849	−0.1108	−0.0816	−0.0720	−0.1282
**N1**		−0.0831			
**N2**			−0.0709		
**N3**				−0.1119	
**C3Sq**					0.0100
**Adj R Sq**	0.5550	0.5597	0.5588	0.5647	0.5597
**Correl Model vs Observed**	0.9816	0.9847	0.9840	0.9881	0.9867
**% MAE Model vs Observed**	3.0%	3.1%	2.7%	2.4%	2.7%
**% MAE Model vs Holdouts**	2.8%	2.8%	3.5%	1.5%	2.6%
**% Residuals >0.05**	11%	13%	21%	11%	13%

## Discussion

The challenges associated with the orthogonal design (inclusion of implausible states and the concentration of states in the moderate range) would have contributed to the relatively low Spearman’s rank correlation coefficients between the results of the ranking task and the ranks of VAS scores of 0.8880 and 0.8884 for the orthogonal valuation subsets (versus 0.94-0.96 for most of the studies that use the MVH protocol). Only 4 respondents in our study (1.7%) preserved the rank order of the states in moving from the ranking task to the VAS task (versus 19% in the MVH study). If it is accepted that the ranking task produces the ordinal preferences of the respondent, then the transfer of cards to the VAS allows the respondent an opportunity to correct any mistakes made during the initial ranking task. Such errors would be most likely among states perceived to be very similar in terms of preference level to the respondent [16]. This is an exploratory study. It was not designed to produce a value set that can be used in resource allocation decision making, but to test the performance of orthogonal valuation subsets and to investigate the impact of modeling and transformation strategies on the utility function. Thus, the respondents used were students because this allowed the convenient creation of two similar respondent groups. Their demographic characteristics and problem rates in Table 1 would not reflect the general population of Trinidad and Tobago. The sample size was also small relative to the sample sizes of VAS studies in the published VAS valuation studies [14]. Despite the small size of the sample, the models in Tables 3 and 4 were all internally valid. Further research could be undertaken using similar studies with larger respondent samples and smaller orthogonal valuation set designs.

This study also demonstrates the performance of VAS as a valuation method for EQ-5D studies, and adds to the literature in support of the VAS as an elicitation instrument [18,19]. Over the last 5–10 years the use of VAS has declined as a means of eliciting health state valuations in EQ-5D studies due partly to a preference for other methods such as Time Trade Off (TTO) and Discrete Choice Experiments (DCE) but also reflecting a criticism of some aspects of VAS methods [20]. One criticism of the VAS is that it is not ‘choice-based’. This criticism has led many researchers away from the method towards choice based approaches such as TTO and DCE. By beginning the VAS valuation with a ranking exercise in which respondents are given the cards one at a time and asked to place each new card in a position based on its level of disutility relative to the other cards in series, this protocol brings ‘choice’ directly into the valuation process. In a cognitive debriefing study of this VAS protocol [21] respondents described the decision making process in the ranking and ranking-to-VAS stages using terms that were virtually identical to their description of their approaches in performing paired comparisons for a DCE. These and other theoretical issues concerning the VAS have been partially dealt with [18,19,22] but there is still resistance to accepting VAS-based valuations in economic evaluation as can be seen in the technical guidance published by national regulatory agencies. Nonetheless, VAS methods are widely used to record consumer preferences in a variety of non-health settings whilst it continues to remain a legitimate method for obtaining the value of self-reported health status—notably as part of the EQ-5D instrument.

## Conclusion

The studies by Lamers [6], and Zarate and Kind [7] suggest that the states that are included in the valuation set have an influence on the model that is obtained in the analysis. In this study, 230 similar subjects (students) divided into two groups each gave VAS valuations of two different sets of EQ-5D states (with no common states between them). When the two data sets were analyzed using the same regression methods, they produced strikingly similar models that performed creditably. This is despite the disadvantages that the orthogonal valuation sets would present (the inclusion of implausible states, and the concentration of states in the moderate range). These encouraging results suggest that further research should be undertaken into using orthogonal array based approaches to developing valuation sets for EQ-5D valuation studies.

This study employed orthogonal arrays with 18 rows (producing valuation sets of 18 states). Further research should be undertaken to test smaller orthogonal designs that can used to produce main effects models. This would allow for smaller samples thus reducing the cost of conducting valuation studies in developing countries. Small orthogonal designs may also permit valuation subsets for TTO studies that do not require blocking, such that each respondent can provide one replicate.

This study found small differences in performance of the models based on data transformed at the level of the individual and at the aggregate level. Differences in performance between the models based on raw VAS data and the models based on transformed data were also very small. The inclusion of the constant term improved the performance of all of the models.

## Endnotes

^a^In Table 1, One Respondent gave their self-reported state as 31111. This is not likely, given the location at which the interviews were conducted. This rating was missed by the interviewer.

^b^In Table 2, the data for one respondent were accidentally lost.
